# Insights into the Biocompatibility and Biological Potential of a Chitosan Nanoencapsulated Textile Dye

**DOI:** 10.3390/ijms232214234

**Published:** 2022-11-17

**Authors:** Eduardo M. Costa, Sara Silva, Freni K. Tavaria, Manuela Pintado

**Affiliations:** Universidade Católica Portuguesa, CBQF—Centro de Biotecnologia e Química Fina—Laboratório Associado, Escola Superior de Biotecnologia, Rua Diogo Botelho 1327, 4169-005 Porto, Portugal

**Keywords:** chitosan nanoparticles, yellow everzol textile dyes, biocompatibility, HaCat cells, antimicrobial activity, skin pathogens, cell infection assays

## Abstract

Traditionally synthetic textile dyes are hazardous and toxic compounds devoid of any biological activity. As nanoencapsulation of yellow everzol textile dye with chitosan has been shown to produce biocompatible nanoparticles which were still capable of dyeing textiles, this work aims to further characterize the biocompatibility of yellow everzol nanoparticles (NPs) and to ascertain if the produced nanoencapsulated dyes possess any biological activity against various skin pathogens in vitro assays and in a cell infection model. The results showed that the NPs had no deleterious effects on the HaCat cells’ metabolism and cell wall, contrary to the high toxicity of the dye. The biological activity evaluation showed that NPs had a significant antimicrobial activity, with low MICs (0.5–2 mg/mL) and MBCs (1–3 mg/mL) being registered. Additionally, NPs inhibited biofilm formation of all tested microorganisms (inhibitions between 30 and 87%) and biofilm quorum sensing. Lastly, the dye NPs were effective in managing MRSA infection of HaCat cells as they significantly reduced intracellular and extracellular bacterial counts.

## 1. Introduction

Synthetic textile dyes have been widely regarded as being hazardous for human health, with carcinogenic and mutagenic effects, and possible toxic effects ranging from contact dermatitis and skin allergies to tumours and heart problems [1,2,3,4,5,6,7,8,9]. Additionally, with the advent of textile functionalization, the need for biologically active molecules has risen and while some natural textile dyes have been reported as possessing some degree of antimicrobial activity, traditional synthetic dyes do not possess such properties and thus an additional antimicrobial coating to be applied to the textile [10,11,12]. Nowadays, alternatives are being sought to improve synthetic dye’s biocompatibility and possibly grant them some biological potential. Among these, alternative nanotechnological approaches have risen to the forefront.

Chitosan (polycationic polysaccharide with high biocompatibility and biological activity)-based nanoparticles (NPs) are one the best candidates for the improvement of synthetic textile dyes as they have proven interactions with dyes [13,14,15]. They also have proven technological viability in the textile industry as they are already used as finishing agents capable of providing antimicrobial activity [16], enhancing the breaking strength, shrink-proofing, adding wrinkle-resistance properties [17,18] and even enhancing textile dyeing [19]. Furthermore, chitosan NPs high loading capacity and ability to limit the compound’s interaction with the external environment leads to reduced compound-mediated toxicity [20].

Considering that a previous work [15] showed that dye nanoencapsulation with chitosan was a viable alternative for the development of novel textile dyeing methodology, while simultaneously producing a nanoencapsulated textile dye which was non-cytotoxic towards human skin cells. A question arose: would it be possible that the nanoencapsulated yellow everzol (NDYE) may have retained the biological potential of the encapsulation molecule (chitosan)? Considering this, we hypothesized that the developed NDYE may be a novel biologically active textile dyeing solution, with relevant antimicrobial and antibiofilm activity against skin pathogens and a capacity to prevent skin cells infection. To prove so, the nanoencapsulated dyes biocompatibility was further characterized, and the biological potential of the yellow everzol NPs against skin pathogens was evaluated through sessile (minimal inhibitory concentration (MIC) and minimal bacterial concentration (MBC) determinations) and planktonic (inhibition of biofilm formation and quorum sensing signalling) assays and through an ex vivo HaCat infection assay.

## 2. Results

### 2.1. Size and Zeta Potential Determination

**Figure 1:**
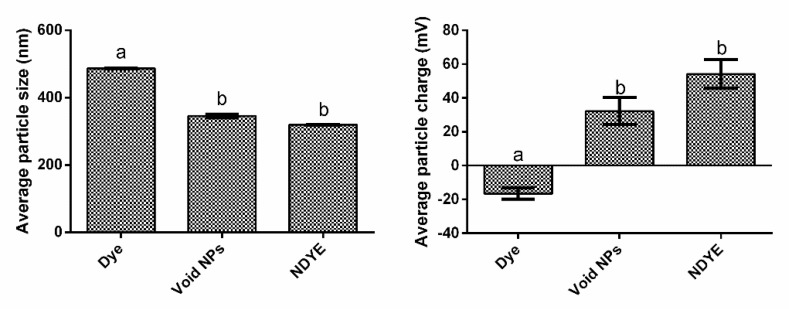
Average particle size (nm) and average particle charge for the free dye, void nanoparticles (NPs) and nanoencapsulated yellow everzol dye (NDYE). Different letters represent the statistically significant (*p* < 0.05) differences found between conditions assayed.

Our results showed that we obtained a well-dispersed suspension of yellow everzol-loaded NPs (Figure 1). The NDYE prepared and analyzed showed an average size of 319.9 ± 0.81 nm with a polydispersity index (PDI) of 0.310. The zeta potential was 54.2 ± 2.14 mV, suggesting that the encapsulation procedure did not affect the positive zeta potential naturally found in chitosan–TPP nanoparticles. In comparison with the free dye and void NPs, it is possible to see that NDYE encapsulation had no statistically significant (*p* > 0.05) effects upon the nanoparticles in comparison with the void NPs but had significant (*p* < 0.05) differences relative to the free dye, especially at the zeta potential level as the free dye had a negative average particle charge.

### 2.2. Biocompatibility Assays

**Figure 2:**
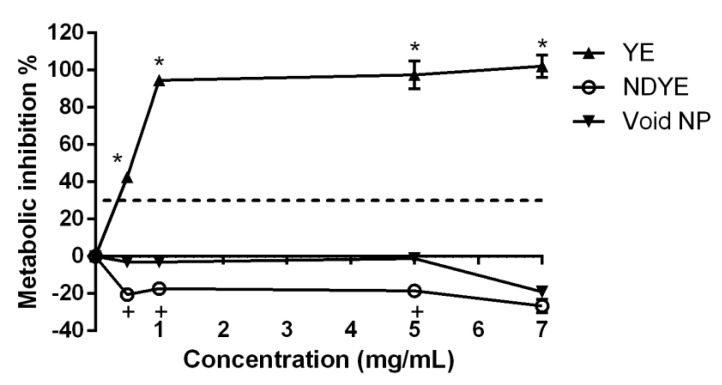
Evaluation of the effect of NDYE (○), void NPs (▼) and yellow everzol (YE) (▲) upon HaCat metabolism, at the different concentrations tested. The dotted line represents the 30% cytotoxicity limit as defined by the ISO 10993-5:2009 standard [21]. * Represents the statistically significant differences (*p* < 0.05) found between the textile dye and the tested nanoparticles. +Represents the statistically significant differences (*p* < 0.05) found between the void and the loaded nanoparticles.

As can be seen from Figure 2 both the void and the loaded NPs tested had no cytotoxic effects upon HaCat cells metabolism at the various concentrations tested.

When considering the activity of the yellow everzol dye on its own the data obtained showed a very high HaCat metabolism inhibition percentage, even at the lowest concentration tested. These values were statistically significantly (*p* < 0.05) higher than those registered for both the NPs loaded with dye and the void NPs. Interestingly enough, barring the higher concentration tested, statistically significant (*p* < 0.05) differences were found between the void and the loaded nanoparticles, with the latter presenting metabolism promotions in all concentrations tested.

**Figure 3:**
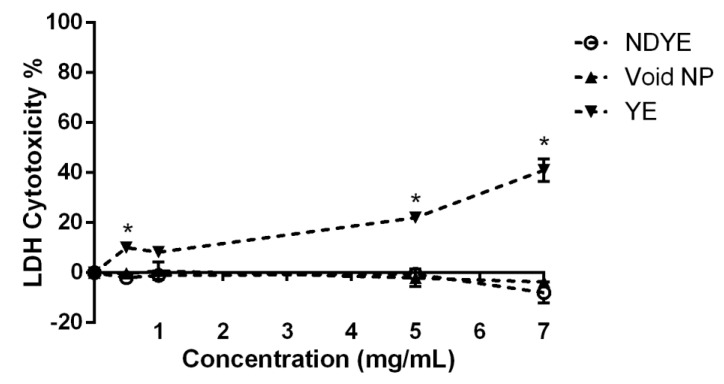
Evaluation of the effect of NDYE (○), void NPs (▼) and yellow everzol (▲) upon HaCat cell wall integrity, at the different concentrations tested. * Represents the statistically significant differences (*p* < 0.05) found between the textile dye and the tested nanoparticles.

When considering the lactate dehydrogenase (LDH) results obtained (Figure 3) it is possible to see that both NPs (void and NDYE) had no cytotoxic effects on HaCat cell walls.

On the other hand, yellow everzol dye on its own showed cytotoxic effects towards the HaCat cells in all tested conditions, with dye toxicity values varying from ca. 10 to 40%. Contrary to what was seen in the metabolism inhibition assay, no statistically significant (*p* > 0.05) differences were observed between NDYE and the void NPs while clear statistically significant (*p* < 0.05) differences were observed between both NPs and yellow everzol dye LDH cytotoxicity levels.

### 2.3. MIC and MBC Determination

The MIC and MBC results obtained for NDYE can be seen in Table 1.

Overall, it stands to notice the lack of MBCs registered for *Pseudomonas aeruginosa* and the low MICs obtained for MRSE and *A. baumannii*. When considering only the MIC results obtained, it’s interesting to see that the average MIC was 1 mg/mL and that no clear patterns of susceptibility were present. When considering antimicrobial resistance as a differentiating factor, antibiotic-resistant strains presented MICs that were either equal (MRSA), superior (VRSA) or inferior (MRSE) to the ones obtained for the sensitive strains (MSSA and *S. epidermidis*). Similarly, when considering the microorganism’s cell wall as a distinguishing factor, the MICs values obtained showed no discernible sensibility patterns that could be observed. Likewise, for the MBC values, no discernible patterns of susceptibility were ascertainable. In fact, excluding the previously referred lack of MBCs registered for *P. aeruginosa*, the other outlier registered was the low MBC observed for MSSA. Furthermore, it also important to reference the low average MBC (2.28 mg/mL) registered and the small difference (1 to 2.28 mg/mL) registered between the average MBC and the average MIC.

### 2.4. Biofilm Formation Inhibition

**Figure 4:**
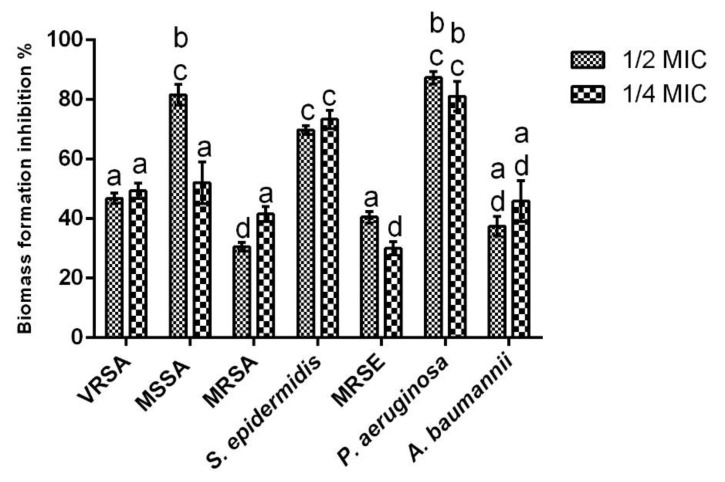
Evaluation of sub-MIC concentrations (½ and ¼ of the MICs) of NDYE upon the selected microorganism’s biofilm formation. Values obtained are given in percentage of biofilm formation inhibition. Different letters represent statistically significant differences found (*p* < 0.05).

As can be seen from Figure 4, the NDYE was capable of having inhibitory activity upon the biofilm biomass formation of the targeted microorganisms.

Overall, all of the inhibition percentages registered were above 30%, with the average inhibition value registered being 54.83% and the highest inhibition obtained being 87.38%. Additionally, it bears notice that for four of the seven microorganisms assayed, the NDYE concentration was not a significant factor, as no statistically significant (*p* > 0.05) differences were found between the tested concentrations. For the cases where statistically significant (*p* < 0.05) differences were found, MRSA inhibition values were a particular case, as the highest inhibition values were registered at the lowest concentration tested. On a closer look, it is interesting to see that for *S. aureus*, the highest sensitivity was registered for MSSA, followed by VRSA and MRSA with inhibition percentages registered for the first being 35 and 40% higher, respectively. A similar pattern of susceptibility was observed for *S. epidermidis* where the antibiotic resistance strain (MRSE) was significantly (*p* < 0.05) less susceptible to NDYE antibiofilm activity. When analyzing the results obtained for the gram-negative strains tested the high inhibition values obtained for *P. aeruginosa* stand out, especially in comparison with the values registered for *A. baumannii*. Interestingly, *A. baumannii* presented a susceptibility pattern similar to MRSA, as the highest biofilm inhibition percentage was obtained at the lowest concentration tested.

### 2.5. Quorum Sensing Inhibition

**Figure 5:**
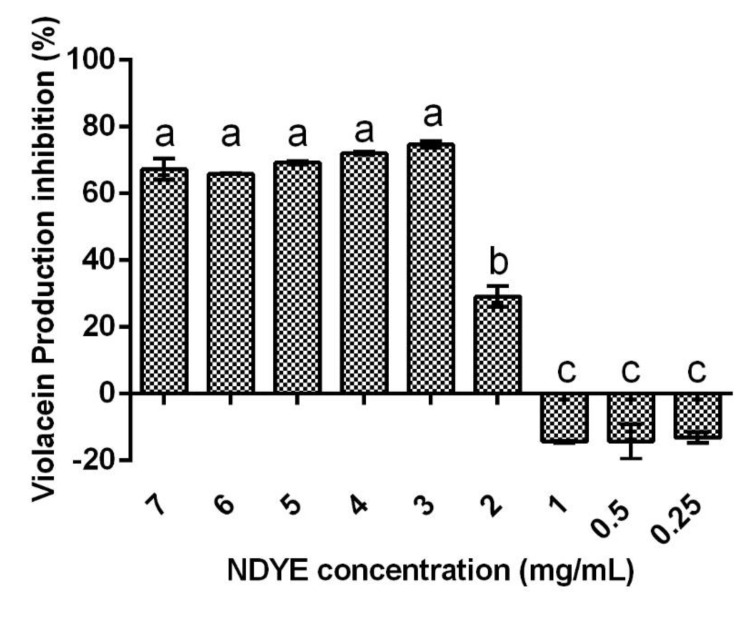
Effect of the tested NDYE concentrations upon the *C. violaceum* quorum sensing reporter system. Results are given in terms of violacein production inhibition percentages. Different letters represent statistically significant differences found (*p* < 0.05).

The quorum sensing inhibition results obtained (Figure 5) show that NDYE inhibited violacein production in the *Chromobacterium violaceum* (*C. violaceum*) reporter system. In fact, for NDYE concentrations between 7 and 3 mg/mL, no statistically significant (*p* > 0.05) differences were found and violacein inhibition percentages varied between 65 and 72%. For concentrations of 2 mg/mL or lower a significant (*p* < 0.05) drop-in activity was observed with violacein inhibition percentages reaching negative values at the lowest concentrations tested.

### 2.6. Cell Infection Assays

**Figure 6:**
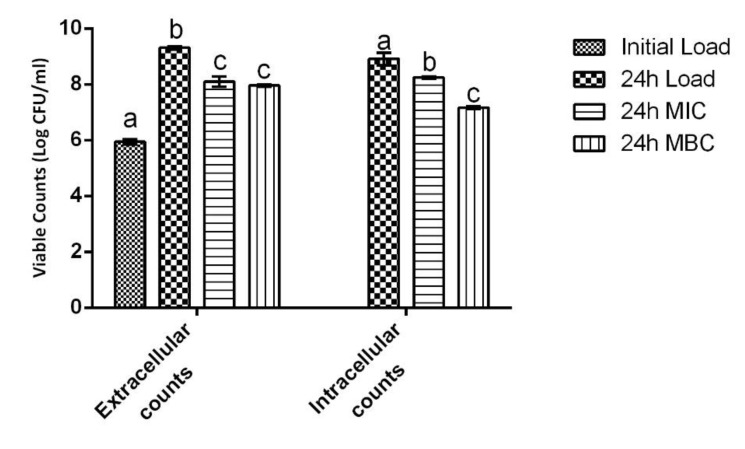
Effect of NDYE, at MIC and MBC concentraions, upon MRSA infection of HaCat cells. Results are given in terms of total bacterial counts. Different letters represent statistically significant differences found (*p* < 0.05) between the tested conditions.

The cell infection results obtained are depicted in Figure 6. As can be seen, statistically significant differences (*p* < 0.05) were found both for the extracellular and intracellular bacterial counts.

When considering only the extracellular counts, NDYE reduced MRSA-viable counts by ca. 1.2 and 1.5 logs of colony forming units (CFUs) at the 24 h mark with no statistically significant (*p* > 0.05) differences being found between the MIC and the MBC. On the other hand, for the intracellular counts, while both concentrations still produced statistically significant (*p* < 0.05) viable counts reductions, the one obtained for the MBC was significantly (*p* < 0.05) higher than the one registered for the MIC. Additionally, it merits to notice the reduced activity that the MIC registered in the intracellular conditions as it only produced a viable count reduction of ca. 0.6 log of CFU versus the 1.2 log of CFU registered in the extracellular settings.

## 3. Discussion

Synthetic textile dyes have been widely regarded as being hazardous to human health, with carcinogenic effects and high cellular cytotoxicity being reported [1,2,3,4,5]. The produced NDYEs presented a relatively small size, low PDI and positive particle charge, all abiding to the intrinsic known characteristics of chitosan NPs [15]. Furthermore, these nanoparticles have been previously shown to be not cytotoxicity towards HaCat cells; these results were further corroborated by the ones registered in this work, as both the XTT and LDH dose–response assays performed here showed that NDYEs did not affect HaCat cell line metabolism and cellular integrity. These results further corroborate the hypothesis advanced by Costa, Silva [15]; dyes entrapment in NPs will block them from interacting with the surrounding environment and thus prevent them from having cytotoxic effects on the HaCat cells.

While some natural textile dyes have been reported as possessing some degree of antimicrobial activity [12], traditional synthetic dyes, like the one used in this work, do not possess such properties and usually either require a modification or an additional application of an antimicrobial coating to the textile [10,11]. Chitosan NPs, on the other hand, have already been established as viable antimicrobial agents as shown in previous works [16,22,23,24,25,26]. However, and considering that, as shown previously [15], the yellow everzol encapsulation process occurs through an interaction of the dyes sulfuric moieties with chitosan amino groups, and it could stand to reason that this could originate loaded chitosan NPs with reduced or non-existent biological activity. As can be seen from the results obtained, this was not the case, as the yellow everzol-loaded NPs were capable of inhibiting the studied microorganisms in all of the different assays performed. Previous works have shown that encapsulating bioactive compounds in chitosan NPs, usually, leads to the formation of compounds with added antimicrobial activity as nanoencapsulation permits compounds to be internalized into cells and thus potentiate their activity [25]. As no previous works have explored the biological potential of nanoencapsulated textile dyes, no direct comparisons were possible. Nevertheless, comparisons, particularly against void chitosan NPs, can still be drawn.

When regarding *S. aureus* and his multidrug-resistant strains (MRSA and VRSA) the existing body of work is twofold. While on the one hand, chitosan NPs have been unanimously shown to be capable of inhibiting the planktonic growth of MSSA [16,23,24,27] (as seen in this work), on the other hand, for MRSA and VRSA of previous works were not as unanimous, with chitosan NPs being described as either capable [22,24,26]) or incapable [28,29,30] of inhibiting MRSA and VRSA. For *S. epidermidis* and MRSE, the obtained results do not find any support in the existing body of work. In fact, the values here reported are drastically different from those reported in previous works, as these have shown that chitosan NPs had either limited activity, as Silva, Silva [29] reported for *S. epidermidis* a MIC of 16 mg/mL, or possessed inhibitory but no bactericidal activity, as reported by Costa, Silva [24] for MRSE. Similarly, the results obtained for *P. aeruginosa* are somewhat contrary to those previously reported. While in previous works [15,31,32,33] chitosan NPs have been described as being effective inhibitors of *P. aeruginosa* growth, the same was not observed here, as no MBC value was registered for the yellow everzol NPs against *P. aeruginosa*. Lastly, for *A. baumannii* the MIC and MBC values obtained are similar to those previously reported by Costa, Silva [26], with the lack of any additional works curtailing any further comparisons. The discrepancies observed between this work and the previous can be justified by the existing literature. The first possible explanation may be related to the particle’s zeta potential, a factor described as being fundamental in NPs antimicrobial activity. As microbial surfaces are negatively charged, NPs with higher zeta potential (as the ones obtained in this work) will have a stronger interaction with the bacteria and thus stronger antimicrobial activity [24,29,34]. Another possible explanation could be related to the NPs quantum size effect, as per this effect, higher antimicrobial activity is associated with smaller particles size, and the particles used in this work are engorged, thus larger, due to the encapsulation of textile dye [25]. Lastly, the last factor affecting the NPs activity may be the microorganisms themselves, because while NPs have been described as possessing no significant differences in sensibility between gram-positive or gram-negative bacteria [35], other microorganism intrinsic factors, such as hydrophilicity and charge density on the bacterial surface, may be at play and thus influence the NPs activity [36]. Additionally, regardless of all of the previous hypotheses, it must not be overlooked that these comparisons are being drawn between void NPs and NPs with altered surface structure (as previously shown by Costa, Silva [15]) due to the yellow everzol entrapment procedure and as such it is possible that any other number of unknown factors may be influencing the observed activity.

Biofilm formation is a crucial process in microbial infection as the majority of infections are attributed to biofilm formation and growth [37,38]. Furthermore, due to their large surface area and hydrophilic porous structure, textiles can retain water, oxygen and nutrients thus creating a propitious environment for microorganism attachment and growth. This attachment is of great concern as textile-embedded bacteria may double every 20–30 min, meaning that one single bacteria cell can increase to 1 × 10^6^ cells in just 7 h [11,39]. As previously established, due to the lack of biological activity of synthetic textile dyes, the obtained results have to be compared to ones previously reported for void NPs. Concerning the activity of the yellow everzol NPs over the staphylococci tested, while previous works are in line with the results obtained here, as void chitosan NPs are reported as being capable of inhibiting MSSA, MRSA and *S. epidermidis* biofilm formation, the same cannot be said regarding MRSE as chitosan NPs are depicted as being incapable of inhibiting MRSE biofilm formation [22,24,40]. Considering that chitosan NPs biological activity is known to be strongly related to its zeta potential [29], and that previously Costa, Silva [24] have hypothesized that MRSE strong production of exopolysaccharides (EPS) during biofilm growth may hamper an NPs capacity to defuse into biofilms structure and interfere with membranes integrity, it is possible that the high zeta potential registered in this work (+54.2 mV to +17.3 mV) may be key in disrupting the biofilms EPS structure and allow NPs to interact with the MRSE cells surface. Regarding *P. aeruginosa* and *A. baumannii*, the obtained results are similar to those previously reported by Costa, Silva [26] for void chitosan NPs, with the higher zeta potential of the particles used in this work (+54.2 mV to +27.1 mV) explaining the higher inhibition percentages obtained here.

Quorum sensing is the way bacteria communicate with each other. By using small, diffusible signals that permit them to modulate various facets of microbial metabolism, such as biofilm formation, the QS molecules and systems present alternative targets for antimicrobial activity [41,42]. The values here registered fall in line with those registered in previous works (76.21%) for chitosan NPs inhibition of violacein production [42].

Methicillin-resistant *S. aureus’s* capacity to invade human host cells is a fundamental part of its infectious process and its capacity to adhere to and infect HaCat cells has been described as being extremely high [43,44,45]. Works exploiting chitosan NPs’, void or loaded, capacity to inhibit bacterial adhesion/infection are few and while none deal directly with textile dye-loaded NPs inhibition of MRSA in HaCat cells, some comparisons are still possible. When concerning chitosan NPs inhibition of *S. aureus* infection of human cells, the results obtained here find purchase in the existing work as Mu, Niu [46] comes in line with the activity here observed as it showed that chitosan conjugated with streptomycin eliminated bacterial intracellular counts in RAW264.7 macrophages. Additionally, when considering chitosan NPs’ capacity to inhibit microorganisms in cellular systems, Mu, Niu [46] in the same work showed that these particles were also capable of eliminating *Listeria monocytogenes* and *Salmonella typhimurium* intracellular counts and that Elbi, Nimal [47] reported that chitosan NPs were capable of reducing, also in RAW264.7 cells, *Salmonella Paratyphi* A intracellular survival. Lastly, as a previous work has shown [26], void chitosan NPs could effectively reduce and inhibit *A. baumannii*’s infection of HaCat cells, thus lending credence to the results here reported.

Overall, the results obtained showed that the nanoencapsulation of textile dyes mitigates their known toxic potential as yellow everzol NPs were completely biocompatible with HaCat cells. Furthermore, yellow everzol NPs were biologically active as they were capable of inhibiting the planktonic and sessile growth of the tested microorganisms and had inhibitory activity in the *C. violaceum* quorum sensing reporter system. Additionally, the NPs were active in an ex vivo setting, as they reduced MRSA extracellular and intracellular counts in HaCat cells. Furthermore, these results open the possibility for the development of bioactive textile dyes and may allow for the elimination of the need for post-dying textile functionalization.

## 4. Materials and Methods

### 4.1. Sources of Chemicals and Solutions Preparation

Low-molecular-weight chitosan (LMW) was obtained from Sigma-Aldrich (St. Louis, MO, USA) and presented a DD between 75 and 85% and an MW of 107 kDa. Sodium Tripolyphosphate (TPP) was purchased from Sigma–Aldrich Chemical Co. Ltd. Reactive (yellow everzol) textile dye was kindly donated by Aquitex S.A. and was prepared at 10 mg/mL using ultra-pure water (Millipore SIM FILTER, Burlington, MA, USA) and stirred until complete dissolution.

### 4.2. Microorganisms and Cellular Line

*Pseudomanas aeruginosa,* VRSA, MSSA and MRSE strains were obtained from the American Type Culture Collection (ATCC 700699, ATCC10145, ATCC 25,923 and ATCC 51,625, respectively; Manassas, VA, USA). *A. baumannii* and MRSA strains were obtained from the culture collection of the Göteburg University (CCUG; Gothenburg, Sweden) (CCUG 61,012 and CCUG 60578). *S. epidermidis* was obtained from the Belgian Co-ordinated Collections of Microorganisms (Belgium) (LMG 10474; Gent, Belgium).

For antimicrobial testing inoculums were prepared in Luria-Bertani Broth (LB; ThermoFisher Scientific, Waltham, MA, USA) and for biofilms assays in Tryptic Soy Broth (TSB, Biokar Diagnostics, Beauvais, France).

For cell-based assays human keratinocytes (HaCat) were obtained from Cell Line Services (Oppenheim, Denmark). For assays cells were cultured, at 37 °C in a humidified atmosphere of 95% air and 5% CO_2,_ using Dulbecco’s Modified Eagle’s Medium (DMEM) with 4.5 g/L glucose, L-glutamine without pyruvate (Lonza, Verviers, Belgium) containing 10% foetal bovine serum (FBS, Biowest, Nuaillé, France) and 1% (*v*/*v*) Penicillin-Streptomycin-Fungizone (Lonza, Verviers, Belgium).

### 4.3. Nanoencapsulated Dyes Production and Characterization

Yellow everzol nanoencapsulation was performed as previously described [15]. Briefly, LMW chitosan was dissolved at 2 mg/mL in acetic acid that was 1.75× more concentrated and the pH was adjusted to 5 with NaOH. TPP was used at chitosan to TPP relation of 7:1. The process began with the addition of 4 mL of chitosan, placed under stirring at 500 rpm, to which a mixture of dye and TPP (1 and 2 mL, respectively) were gently added dropwise, at room temperature. At the end of the process, the loaded NPs were frozen and freeze-dried for posterior use in the biological assays.

The prepared dyed loaded NPs physical properties were analyzed through dynamic light scattering (DLS) using a Malvern Instruments NanoZSP (Worcestershire, UK) with particle size (PS), polydispersity index (PDI) and zeta potential (ZP) being determined. All assays were performed using a disposable folded capillary cell (Malvern, Worcestershire, UK), with a 90° laser angle, at room temperature (25 °C). Size values were determined based on intensity distribution. All assays were performed in sextuplicate.

### 4.4. Biocompatibility Assays

#### 4.4.1. XTT Assay

The produced nanoparticles’ impact upon HaCat metabolism was performed as previously described using the XTT viable dye [15,48]. Briefly, HaCat cells were seeded at 1 × 10^5^ cells/mL in the wells of a 96-well microplate and allowed to adhere. After 24 h, the media was removed, and the cells were washed with PBS. Following this, media with loaded and void NPs (7 mg/mL to 0.5 mg/mL) was added. Simultaneously, two controls were accessed: one with textile dyes at 7 mg/mL and the other with sterile water. After 24 h, 25 µL of XTT working solution were added to each well and the cells were incubated, in the dark, for 2 h. The optical density (OD) at 485 nm was then measured using a microplate reader (FLUOstar, OPTIMA, BMG Labtech, Ortenberg, Germany). All assays were performed in quintuplicate.

#### 4.4.2. LDH Leakage Assay

Nanoparticles cytotoxicity was assessed by LDH leakage into the culture medium. Following the exposure of HaCat cells to void and loaded nanoparticles (7 mg/mL to 0.5 mg/mL), the activity of LDH in the medium was determined using the commercially available kit from ThermoScientific (Rockford, IL, USA) and using accordingly to the manufacturer’s instructions. Briefly, cells were seeded at 2 × 10^4^ cells/100 µL of media, allowed to adhere for 24 h and then exposed to the nanoparticles. After 24 h incubation cells were processed accordingly to the kits instructions and the results were given accordingly to the following equation:

% Cytotoxicity = [(Compound treated LDH activity − Spontaneous LDH activity)/(Maximum LDH activity − Spontaneous LDH activity)] × 100(1)

### 4.5. Antimicrobial Activity

Evaluation of the compounds’ MIC was executed as previously described Silva, Costa [49]. Briefly, a 0.5 MacFarland inoculum of each microorganism was prepared and inoculated in Muller Hinton Broth (Biokar Diagnostics, Beauvais, France) with dye loaded NPs concentrations ranging from 0.1 to 7 mg/mL. Simultaneously, a negative (non-inoculated media with NPs at 0.1 mg/mL) and a positive control (inoculated media with microorganism only) were also evaluated. The MIC was determined by observing the lowest sample concentration at which no bacterial growth was visible. All assays were performed in triplicate. Determination of the MBC was performed as previously described by Costa, Silva [26]. Succinctly, MBCs were defined as the lowest sample concentration at which bacterial growth in agar plates was prevented and it was evaluated by inoculation of 100 µL aliquots of negative wells (absence of turbidity in MIC determination) in Plate Count Agar (PCA, Biokar Diagnostics, Beauvais, France), using the plate spread technique. All assays were performed in quadruplicate.

### 4.6. Antibiofilm Activity

Dye-loaded NPs’ capacity to inhibit biofilm formation was evaluated as previously by Costa, Silva [50]. Briefly, in a flat-bottom 96-well microplate, TSB supplemented with 1% (*w*/*v*) glucose and dye NPs at sub-MIC concentrations (1/2 and 1/4 of the MIC) (was inoculated at 2% (*v*/*v*) using an overnight inoculum and was incubated for 24 h at 37 °C. Afterwards, the contents of the wells were discarded and each well was washed to remove non-adhered cells. Quantification of biofilm formation was performed through determination of the biomass present using the crystal violet assay. All assays were performed in quadruplicate.

### 4.7. Quorum Sensing (QS) Inhibition Assay

Screening of the QS inhibitory activity of NDYE was carried out based on their ability to inhibit the production of the purple pigment violacein by *C. violaceum*. The assay was performed as described by Costa, Silva [51]. Briefly, in a 96-well microplate, wells were filled with 200 mL of the test solution with NDYE added at concentrations between 7 and 0.25 mg/mL. Simultaneously, a positive control, where chitosan was substituted with water, was also assayed. Following that, the plate was incubated at 37 °C for 24 h in a microplate reader (FLUOstar, OPTIMA, BGM Labtech) with OD being measured at 577 nm for violacein production and 660 for bacterial growth. All assays were performed in triplicate.

### 4.8. Cell Infection Assays

The dyes NPs’ antimicrobial in a cellular system was evaluated via adaptation of the work previously published by Pati, Mehta [52]. Briefly, human keratinocytes were seeded at 1 × 10^5^ cells/mL and allowed to adhere. After 24 h, the media was removed, cells were washed with PBS and then three conditions were assayed: (i) cell culture media with MRSA (1 × 10^6^ CFU/mL) and NDYEs at MIC; (ii) cell culture media with MRSA and NDYEs at MBC; (iii) cell culture media with MRSA only (infection control). After 24 h, the extracellular and intracellular viable counts were determined through serial dilution and plating. For the extracellular counts, the supernatant from the wells was harvested while for the intracellular counts HaCat cells were lysed with triton, harvested with PBS and then processed for viable counts. For both conditions, viable counts were determined by the drop method as previously described by Costa, Silva [26]. Results were given for both assays as total viable counts (extracellular counts) and total bacteria by HaCat cell (intracellular counts). All assays were performed in quadruplicate.

### 4.9. Statistical Analysis

This work’s statistical analysis was performed using IBM SPSS Statistics v21.0.0 (New York, NY, USA) software. Considering that data followed a normal distribution, a one-way ANOVA coupled with Turkey’s post hoc test was performed with Turkey’s post hoc test being used with differences being considered significant for *p*-values below 0.05. Graphical processing of the data was performed using Graphad Prism 6 software (San Diego, CA, USA).

## Figures and Tables

**Table 1:** MIC and MBC values obtained for NDYE against the target microorganisms. All results are given in mg/mL of NDYE. nd—not determined.

Microorganism	MIC	MBC
Methicillin-sensitive *Staphylococcus aureus*	1	1
Methicillin-resistant *Staphylococcus aureus*	1	3
Vancomycin-resistant *Staphylococcus aureus*	2	3
*Staphylococcus epidermidis*	2	3
Methicillin-resistant *Staphylococcus epidermidis*	0.5	3
*Pseudomonas aeruginosa*	1	Nd
*Acinetobacter baumannii*	0.5	3

## Data Availability

The data presented in this study are available on request from the corresponding author. The data are not publicly available due to confidentiality agreements.
